# Self-management of HTLV-1-associated myelopathy/tropical spastic paraparesis in Japan: A qualitative study

**DOI:** 10.1371/journal.pntd.0014055

**Published:** 2026-03-19

**Authors:** Saori Yamaguchi, Rika Yatsushiro

**Affiliations:** Department of Nursing, School of Health Sciences, Faculty of Medicine, Kagoshima University, Kagoshima, Japan; Public Health Agency of Canada, CANADA

## Abstract

**Background:**

HTLV-1 associated myelopathy/tropical spastic paraparesis (HAM/TSP) is a spastic spinal cord-related paralysis that is caused by a human T-lymphotropic virus type 1 infection. Common symptoms of HAM/TSP include gait disorder, bladder disturbance, constipation, and sensory symptoms. Because no curative treatment exists, individuals with HAM/TSP have to self-manage their chronic condition. Although patients with HAM/TSP have unique self-management strategies owing to their complex backgrounds, limited information is available on how they self-manage their chronic conditions. This study aimed to explore the self-management strategies of patients with HAM/TSP in Japan.

**Methods:**

Using semi-structured interviews and an open-ended questionnaire, data were collected from seven participants with HAM/TSP. Interviews were transcribed verbatim. Data analysis was conducted using a qualitative content analysis method to identify categories that describe the self-management strategies of people with HAM/TSP.

**Findings:**

Six core categories of self-management strategies of people with HAM/TSP were identified: “Attempting to maintain body functions”, “Acquiring new ways to complement body functions”, “Engaging to maintain an independent daily life”, “Ensuring a specialized treatment environment for HAM/TSP”, “Taking control of HAM/TSP”, and “Moving forward as people with HAM/TSP.”.

**Conclusions:**

The self-management strategies of people with HAM/TSP are affected by three characteristics of HAM/TSP: the lack of an established curative treatment, the low social awareness of the disease, and its infectious nature. This study highlights the need for personalized educational strategies and accessible support for the development of self-management skills.

## Author summary

HTLV-1-associated myelopathy/tropical spastic paraparesis (HAM/TSP) is a spastic spinal cord-related paralysis caused by the human T-lymphotropic virus type 1 infection. It is estimated that 3000 individuals in Japan have HAM/TSP, which presents with diverse symptoms that affect daily life. As HAM/TSP lacks an established curative treatment, patients must self-manage the impact of HAM/TSP on their daily lives. To facilitate informed nursing support that enables HAM/TSP self-management, in this study, we identified 6 self-management strategies of people with HAM/TSP: “Attempting to maintain body functions”, “Acquiring new ways to complement body functions”, “Engaging to maintain an independent daily life”, “Ensuring a specialized treatment environment for HAM/TSP”, “Taking control of HAM/TSP”, and “Moving forward as people with HAM/TSP.” Although the results of this study may not describe all aspects of self-management in patients with HAM/TSP, the findings provide an overview of self-management strategies and variations that constitutes foundational research findings in this area. This study suggests the necessity of personalized educational strategies and accessible support for the development of self-management skills.

## Introduction

Chronic conditions, including communicable and noncommunicable diseases, certain mental disorders, and ongoing physical impairments, constitute persistent health problems that require some degree of healthcare management. Individuals with chronic conditions need to learn how to self-manage the effects of their illness over the long term [1], using strategies that must be incorporated into their daily routine. To assume new responsibilities related to the self-management of chronic conditions, individuals must actively partner with healthcare professionals [2].

HTLV-1-associated myelopathy/tropical spastic paraparesis (HAM/TSP) is a spinal spastic paraparesis that is caused by a human T-lymphotropic virus type 1 infection. Osame et al. proposed HAM as a new clinical entity in Japan [3], and Gessain et al. demonstrated HTLV-1 seropositivity of patients with TSP in the Caribbean [4]. The results of additional studies confirmed that HAM and TSP were the same clinical entity and, in 1988, the World Health Organization named this condition HAM/TSP [5]. HTLV-1 is transmitted by breastfeeding, sexual intercourse, blood transfusion, and needle sharing; approximately 10 million people worldwide are HTLV-1 carriers, of whom between 0.1% and 2% have HAM/TSP [6,7]. In Japan, approximately 3000 individuals have been diagnosed with HAM/TSP. Typical symptoms of HAM/TSP include chronic spastic paraparesis-induced gait disorders, weakness of the lower limbs, bladder disturbance, constipation, and sensory symptoms, such as tingling, pins and needles, and burning [8]. Treatment is available for the reduction of spinal inflammation caused by corticosteroids and interferon-α; however, there is currently no curative treatment for HTLV-1 itself [7]. For symptoms such as lower-limb spasticity and bladder dysfunction, symptomatic treatment with drugs is administered. Without no established curative therapeutic strategy, individuals with severe HAM/TSP must change not only their daily behaviors but also their entire way of life. Our previous study found that people with HAM/TSP experienced a “feeling of loss of self-control” over their body, HAM/TSP as a disease, and their future during the progression of symptoms. It is important to support the attempts of these individuals to manage the problems caused by HAM/TSP as a chronic condition and provide an opportunity for self-management of persistent symptoms as this may help them find positive meaning in their illness and improve their quality of life.

Self-management has gained importance in chronic illness care [9] and is defined as “learning and practicing skills necessary to carry on an active and emotionally satisfying life in the face of a chronic condition” [10]. Self-management science has developed partly because acute care models do not satisfy the needs of people with chronic conditions [1]. Research has already described and evaluated self-management interventions in various areas and confirmed the effectiveness of self-management programs in participants who significantly improved their self-management behaviors [11]. The self-management approach is effective for supporting not only lifestyle-related diseases but also progressive neurological disorders, and the Taxonomy of Everyday Self-management Strategies (TEDSS) was developed as one of the few self-management frameworks from the perspective of people living with neurological conditions [12]. The most common neurological disease studied in the context of self-management is multiple sclerosis (MS) [13], and a systematic review showed evidence that supports the value of programs designed to promote self-management of MS and other neurological diseases [14]. Considering the common symptoms, uncertainty of disease course, and progressive disability, research on self-management in MS is very informative for individuals with HAM/TSP; therefore, we reviewed published studies on self-management of MS to explore the significance and potential of self-management for people with HAM/TSP [15]. The results showed that a detailed investigation into self-management of people with HAM/TSP from the patient’s perspective is required to develop effective self-management interventions that reflect the often unique nature of the disease for each individual.

According to Lorig and Holman, although many concerns are shared across different diseases, behaviors, and populations, there are intergroup and interindividual differences in the needs [11]. The best self-management support for people with neurological disorders should go beyond standard advice and must be customized to each individual’s reality, life context, and disease trajectory [16]. People with HAM/TSP have unique experiences owing to their complex backgrounds; furthermore, the number of patients is small, there is no cure, the disease is infectious, and it can manifest with diverse symptoms. Specifically, HAM/TSP is not socially recognized and is likely to be misunderstood because of the interindividual variations in symptoms. Moreover, because HAM/TSP is an infectious disease, stigma toward viral infections may arise. A recent study about the lived experience of people with HAM/TSP in Iran found that they had “reduced self-sufficiency and social dignity,” which included three main categories “disruption of desirable personal and social life,” “reduced perception of role competencies,” and “obligatory unpleasant lifestyle changes” [17]. However, to the best of our knowledge, no research on the self-management of patient with HAM/TSP in Japan has been reported thus far. There is limited information regarding the process by which individuals with HAM/TSP self-manage their chronic health condition. Consequently, nursing care may not reflect the opinions of the patients with HAM/TSP. Therefore, the investigation of self-management among people with HAM/TSP is a priority. Accordingly, this qualitative study aimed to explore the self-management strategies of individuals with HAM/TSP in Japan.

## Methods

### Ethics statement

This study was approved by the Ethics Committee for Epidemiological Research of the Kagoshima University (approval number: 80267(379) epi-ver. 3). The necessary information about the study was explained in writing to patients with HAM/TSP by the researcher, and written informed consent was obtained from individuals with HAM/TSP who voluntarily agreed to participate in this study.

### Study design

A qualitative descriptive research approach was used because we aimed to provide a better understanding of self-management and contribute to building knowledge based on the experiences of patients with HAM/TSP. The qualitative descriptive research approach is based on naturalistic inquiry and is suitable for describing a comprehensive summary of an event in everyday terms from an insider’s perspective [18,19].

### Participants

The data for this study were based on the narratives of personal experiences of patients with HAM/TSP. Therefore, the inclusion criteria were as follows: individuals older than 20 years with HAM/TSP and able to communicate verbally, diagnosed with HAM/TSP, and recuperating at home for at least a year in total since diagnosis, without specifying whether they were receiving treatment for HAM/TSP. For the recruitment of research participants, we sought the cooperation of representatives of non-profit organizations to which the HAM/TSP patient association is affiliated. Leaflets seeking potential research participants were distributed to members of the patient associations, and the first author contacted each patient group member who expressed an interest to participate in the study to confirm their willingness to participate and arranged a date and time for an interview.

### Data collection

Data were collected using semi-structured interviews and an open-ended questionnaire. Interviews were conducted by the first author, who had prior experience interviewing more than 10 patients with HAM/TSP in previous studies. Interviews were conducted using an interview guide based on the research questions. Demographic information and HAM/TSP symptoms were recorded at the beginning of the interviews. The questions in the interview guide were as follows: 1) Please tell us about the health problems caused by HAM/TSP and the difficulties you have in living with them in your daily life and 2) What kind of regimen do you use to manage and control the health problems and difficulties caused by HAM/TSP in your daily life? 3) How do you incorporate the ways in which you manage and adjust yourself to the health problems caused by HAM/TSP and the associated difficulties in living with them in your daily life? 4) What factors do you feel influence your decision to choose and implement ways to manage or adjust yourself to the health problems caused by HAM/TSP and the associated difficulties in living with it? 5) What do you feel means you can manage and adjust your own health problems caused by HAM/TSP and the associated difficulties in your life? 6) How have managing or adjusting to your own health problems or difficulties caused by HAM/TSP affected you and your life? Please describe this outcome.

Interviews were conducted at the participant’s home or in a private room where the participant’s privacy was ensured. An interview with a duration of approximately 1 hour per participant was planned, and a second interview would be planned if additional data collection was necessary. The interviews were recorded with the consent of the participants and the recordings were transcribed verbatim. Observations and informal interviews conducted during, before, and after the interviews were recorded in field notes with the consent of the research participants and then used as a reference for data analysis. After the interviews were completed, the participants were asked to complete an open-ended questionnaire consisting of one question, “Please tell us about any health problems or difficulties in your life that are caused by HAM/TSP and how you adjust or self-manage them yourself. Please feel free to provide any additional information below that you would like to add or supplement what you told us in the interview.” The questionnaire was given to them along with a return envelope in order to supplement the interview content and provide information about the participants’ self-management, which was not discussed in the interview. These questionnaire responses were included in the analysis.

### Data analysis

Data analysis was conducted using qualitative content analysis [18,19]. Unlike quantitative content analysis, where researchers systematically apply existing codes to data, the qualitative content analysis involves the generation of codes from the data itself during the analysis [18].

First, the researchers carefully read the data collection-derived descriptions of self-management several times. Thereafter, the descriptions were separated based on the meaning of the content and each section was coded without compromising their semantic content. The codes were organized by comparisons of any similarities and differences, and we extracted subcategories that were compared for similarities to further extract categories; finally, the core categories were extracted.

To collect data, a triangulation of two methods – semi-structured interviews and open-ended questionnaires – was used to ensure the credibility of the data on self-management in people with HAM/TSP. At the end of their interview, we asked the participants whether they had anything to add and they reviewed the summary of the interviews by the researcher. Although the principal researcher interviewed more than ten people with HAM/TSP in previous studies, interviewer attitude and interviewing techniques were reviewed to ensure interviewing competence. The data collection and analysis processes for this study were documented in detail, leaving a trail for audit, and the trustworthiness of the results of the analysis was ensured by member checking of the data analysis by another researcher.

## Results

Seven people with HAM/TSP who consented to participate in the study were interviewed once each, and each interview lasted 58–134 (average, 80) min. No participant required a second interview. After the interviews, three participants returned the open-ended questionnaire; thus, the descriptions were treated as data. Of the seven participants, four were male and three were female, and the median age of the cohort was 71 (IQR, 66.5–73) years. Here, participants were recruited directly through the HAM/TSP patient association, and thus, we were unable to access participants’ medical records. Therefore, the diagnostic process leading to the HAM/TSP diagnosis, including any past or ongoing treatments, were confirmed through interviews with the participants themselves. The participants’ diagnosis of HAM/TSP was confirmed during interviews based on their statements that they had undergone cerebrospinal fluid testing for diagnosis, had applied to the government for medical care of patients with intractable diseases as HAM/TSP patients, and had participated in HAM/TSP clinical trials. The median duration since HAM/TSP diagnosis was 15 (IQR, 13–18) years. All but one participant had received corticosteroid or interferon-α therapy. Two participants were taking muscle relaxants such as baclofen, one participant was receiving drug therapy for urinary frequency, and two participants were taking alpha-1 blockers for voiding symptoms. The Osame Motor Disability Score (OMDS) [20], which assesses motor disability in HAM/TSP from 0 (normal walking and running) to 13 (complete bedridden), ranged from 4 (need support while using stairs) to 10 (crawls with hands), and four participants with OMDS scores of 6 or higher used a wheelchair for mobility. Six participants had bladder dysfunction and one required intermittent self-catheterization. In addition, six participants each had bowel dysfunction and sensory disturbance. To ensure confidentiality and prevent patient identification, the individual characteristics of the participants (Participant A to Participant G) will not be published.

From an analysis of the data, we identified 6 core categories, 14 categories, and 39 subcategories as self-management strategies that involved activities to manage their chronic conditions caused by HAM/TSP (Table 1). The six core categories were: “Attempting to maintain body functions”, “Acquiring new ways to complement body functions”, “Engaging to maintain an independent daily life”, “Ensuring a specialized treatment environment for HAM/TSP”, “Taking control of HAM/TSP”, and “Moving forward as people with HAM/TSP.” Each core categories, categories and subcategories are explained briefly with illustrative quotations from the study participants below.

**Table 1:** Core Categories, Categories and Subcategories.

Core Categories	Categories	Subcategories
Attempting to maintain body functions	Attempting to control own excretion	Seeking ways to control urination Excreting residual urine according to the rule of thumb Undertaking efforts to maintain bowel movement Understanding the effects of laxative Self-adjusting laxative dosage regimen
	Training to maintain motor function	Recognizing the need for physical activity Training to maintain motor function
Acquiring new ways to complement body functions	Incorporating new ways to maintain excretion	Understanding the need for self-catheterization Adopting excretory new ways to maintain excretory function Choosing a way of dealing with excretion does not affect daily life
	Acquiring an alternative means of mobility to walking	Recognizing the need to consider alternatives Replacing walking with a wheelchair Acquiring the means to drive manually
Engaging to maintain an independent daily life	Seeking ways to maintain independence	Doing what I can by myself Improving the environment to maintain independence Developing ways to maintain independence Dealing with what might happen in advance
	Receiving the required support	Approaching to get the official support needed Receiving official support Receiving support from family and surroundings Realizing be understood by those around one
Ensuring a specialized treatment environment for HAM/TSP	Ensuring medical treatment by HAM/TSP specialist	Maintaining connections with HAM/TSP specialist Ensuring medical treatment by HAM/TSP specialist Continuing to see HAM/TSP specialist
	Supporting research on the treatment of HAM/TSP	Cooperating with research activities related to HAM/TSP Enrolling in the HAM/TSP clinical trial Expecting to develop a treatment of HAM/TSP
Taking control of HAM/TSP	Acting to understand HAM/TSP	Gathering information about HAM/TSP Connecting with people associated with HAM/TSP
	Managing the medical conditions of HAM/TSP	Confirming medical conditions of HAM by blood tests Taking care not to worsen the medical condition
	Self-determination regarding treatment	Receiving drug treatment Making the choice not to receive drug treatment Self-evaluating one’s choices regarding treatment
	Concern about the impact of HTLV-1 infection on close relatives	Concern about the impact of HTLV-1 infection on close relatives
Moving forward as people with HAM/TSP	Supporting each other among people with HAM/TSP	Digesting negative emotions with people with HAM/TSP Supporting people with HAM/TSP as a fellow patient
	Accepting the reality of what HAM/TSP brings	Accepting living with HAM/TSP positively Not resisting the reality that HAM/TSP brings

### Attempting to maintain body functions

People with HAM/TSP attempted to maintain HAM/TSP-affected body functions by trying to control their excretory functions and undertook training to maintain motor function. Considering the lack of an established cure for HAM/TSP, participants recognized the need to self-manage the primary symptoms of HAM/TSP-gait disorder, bladder disturbance, and constipation, and thus attempted to maintain their own functions through various ways.

#### Attempting to control own excretion.

Attempts to control bladder dysfunction are described by the strategies “Seeking ways to control urination,” and “Excreting residual urine according to the rule of thumb.” Participants engaged in “Seeking ways to control urination” by seeking medication, reducing fluid intake, and other methods. In addition, through their own experiences, they found strategies, such as stimulating the bladder to expel urine, and they performed “Excreting residual urine according to the rule of thumb.”

“When I was about to urinate, it was not vigorous and only came out in drops, so I would push on the bladder like this… Then, you know, urine comes out increasingly.” (Participant G).

Attempts to control bowel dysfunction are described by the strategies “Undertaking efforts to maintain bowel movement,” “Understanding the effects of laxatives,” and “Self-adjusting laxative dosage regimen.” Participants reported “Undertaking efforts to maintain bowel movement” by means other than drugs, such as food and exercise. In addition, the participants for whom laxatives were effective established “Self-adjusting laxative dosage regimen,” with the permission of their physicians to adjust the dosage and timing of their prescribed laxative doses based on “Understanding the effects of laxative.”

“Yes, I’m not taking any medicine. Well, I am currently taking things like prunes and tea that help with bowel movements and trying to go as naturally as possible.” (Participant A).

“If there is no bowel movement for two or three days, I increase the dosage to three pills and adjust the dosage and intervals flexibly according to the situation.” (Participant C).

#### Training to maintain motor function.

Individuals with HAM/TSP were consciously trained to maintain their motor function. “Recognizing the need for physical activity” refers to the recognition of the need to intentionally move or rehabilitate the body to maintain muscle strength and stop the progression of gait disturbances. “Training to maintain motor function” refers to receiving regular professional rehabilitation or performing independent exercise.

“I think that doing whatever strength training I can do, even if it’s not at a fitness club is an important thing for me to stay current.” (Participant E).

“You know, I think I will not be able to move. When I was first hospitalized with HAM, I felt the most that I would not be able to move. So I am doing my rehabilitation to the death.” (Participant F).

### Acquiring new ways to complement body functions

Patients with HAM/TSP who experienced significant disability in their body functions choose to incorporate new strategies to maintain their excretory and motor functions. This strategy was employed during the insufficiency of self-management strategies for maintaining their physical functions. As there is no curative treatment for HAM/TSP, new approaches are needed to manage progressive functional disability.

#### Incorporating new ways to maintain excretion.

Patients with HAM/TSP have incorporated new ways to maintain excretory function, such as self-catheterization and enemas, as well as coping strategies to ensure that excretion does not affect their daily lives. “Understanding the need for self-catheterization” refers to the understanding that impaired bladder function with residual urine after urination requires self-catheterization. “Adopting excretory new ways to maintain excretory,” such as self-catheterization and enema, was a strategy to maintain elimination. “Choosing a way of dealing with excretion does not affect daily life,” such as sleeping close to a bathroom, using a bedpan, and using diapers and incontinence pads for unexpected urine leakage, were also coping strategies that were practiced by the people with HAM/TSP to maintain elimination.

“When I told my doctor that I had a feeling of residual urine, he explained to me that I should perform self-catheterization just once a day to empty my bladder, so I started.” (Participant C).

“After I’ve leaked urine, I used only incontinence pads at first, but I thought that pull-up diapers would be better than pads, so I use diapers like this, so that relief? “(Participant A).

#### Acquiring an alternative means of mobility to walking.

As people with HAM/TSP approached the stage where they had trouble walking because of progressive motor disability, they recognized the need for alternative means of mobility besides walking and, depending on their individual conditions, acquired wheelchairs or a means to enable manual driving. “Recognizing the need to consider alternatives” refers to recognizing the need to change to new means when they can no longer move around on their own. “Replacing walking with a wheelchair” and “Acquiring the means to drive manually” refer to the use of a wheelchair as an alternative to walking and the acquisition of a manual driving device as an alternative to driving a foot-operated vehicle, respectively.

“I still think there is a need to make changes in response to the inability to drive, for example, by using cabs.” (Participant A).

“I thought, Oh, well, I can do it if I drive by hand, So I bought a new car with a manual driving system, and even now I drive manually.” (Participant B).

### Engaging to maintain an independent daily life

A self-management strategy employed by people with HAM/TSP involves seeking ways to maintain independence and receiving the required support, and this enables them to lead independent daily lives despite HAM/TSP’s effect on their body functions. Similar to the two self-management strategies that have been mentioned above, the lack of established treatments for HAM/TSP necessitates the exploration of strategies to live independently despite physical functional disability. For availing official or informal support, the participants needed to seek out support by themselves and this necessitated that they be understood by those around them; this requirement indicates the impact of low social awareness of HAM/TSP on the self-management of individuals with HAM/TSP.

#### Seeking ways to maintain independence.

People with HAM/TSP sought ways to maintain their independence as their HAM/TSP symptoms progressed and they became increasingly unable to do more things in their lives alone. “Doing what I can by myself” describes a behavior that includes the intention of people with HAM/TSP for independence. “Improving the environment to maintain independence” and “Developing ways to maintain independence” refer to activities of the people with HAM/TSP improving their physical environment or developing ways to do things on their own to maintain independence. “Dealing with what might happen in advance” refers to actions preparing for and dealing with what might happen, either with current symptoms or with the progression of symptoms.

“I think that it can’t be helped what I can’t, but I will do what I can....I have a strong wish to do what I can do because it’s my own thing.” (Participant D).

“Well, we renovated everything with the expectation that I would be on a wheelchair in the future.” (Participant C).

#### Receiving the required support.

People with HAM/TSP approached and used available official support and informal support from family members and others. “Approaching to get the official support needed” refers to actions undertaken to identify the available official support and to apply for it, and “Receiving official support” refers to the use of public support. “Receiving support from family and surroundings” refers to receiving the necessary support from family and others appropriate to their situation, and “Realizing to be understood by those around one” refers to the realization that the disease condition of HAM/TSP and their health situation as it related to HAM/TSP are understood by those around them.

“I asked the Community Comprehensive Support Center to come and certify my long-term care insurance, and at that time, I was at Support Care Level 2. From the beginning (since HAM/TSP was added as a designated incurable disease), I applied for certificates issued for specific diseases (application for medical care of patients with intractable diseases).” (Participant G).

“My neighbors all know that I have this disease, and in that sense…I think I have gained their understanding of my illness by speaking out about it increasingly.” (Participant B).

### Ensuring a specialized treatment environment for HAM/TSP

Ensuring specialized medical treatment for HAM/TSP and cooperating to develop curative treatments are important self-management strategies for people with HAM/TSP to ensure the creation of an appropriate therapeutic environment. Participants understood that accessing care from HAM/TSP specialists is difficult, to the extent that even diagnosis of HAM/TSP can be time-consuming. Ensuring medical treatment by HAM/TSP specialists was identified as a self-management strategy. In the absence of a curative treatment for HAM/TSP, initiatives such as supporting physicians’ research activities and participating in clinical trials for HAM/TSP treatments could contribute to improve the treatment environment for HAM/TSP patients, which further advances self-management.

#### Ensuring medical treatment by HAM/TSP specialist.

People with HAM/TSP are eager to be seen and treated by HAM/TSP specialists, and they act to ensure this. For people with HAM/TSP who do not have a HAM/TSP specialist in the neighborhood, “Maintaining connections with HAM/TSP specialist” and “Ensuring medical treatment by HAM/TSP specialist” were an important task. Therefore, if they were in a situation where they could receive medical care from a HAM/TSP specialist, they undertook the action of “Continuing to see HAM/TSP specialist”.

“I contacted members of the XX Patients’ Association when the doctor was coming here, and we got together. The doctor would hold a meeting two or three times a year where everyone could consult with the doctor. Since the opening of outpatient clinics, we do not have to go to XX (where the specialists are located), but we can all see this here.” (Participant B).

#### Supporting research on the treatment of HAM/TSP.

As no curative treatment for HAM has been established, participants with HAM/TSP actively encouraged research on the treatment of HAM/TSP by “Cooperating with research activities related to HAM/TSP” and “Enrolling in the HAM/TSP clinical trial.” “Expecting to develop a treatment of HAM/TSP” was the driving force behind their actions.

“I would like to continue to actively participate in clinical trials in the hope of establishing a treatment for this disease as soon as possible.” (Participant G).

### Taking control of HAM/TSP

People with HAM/TSP took control of their HAM/TSP by actively engaging in understanding HAM/TSP, managing their condition, and making their own decisions regarding treatment. In the context of the low social recognition and unfamiliarity with HAM/TSP even among the participants themselves, acting to understand HAM/TSP was identified as a self-management strategy. Considering the lack of a curative treatment, participants carefully made their own decisions regarding the treatment that was proposed by their physicians. Furthermore, because HAM/TSP is an infectious disease, concern about the impact of HTLV-1 infection on close relatives constituted an aspect of the self-management strategy.

#### Acting to understand HAM/TSP.

To understand the disease, people with HAM/TSP engaged in actions to gather information about HAM and connect with others, such as by participating in patient associations. “Gathering information about HAM/TSP” refers to actions that were intentionally undertaken to gather information about HAM/TSP and “Connecting with people associated with HAM/TSP” refers to making direct connections with doctors and people with the same disease to understand HAM/TSP.

“I wanted to get more real information, so we formed the branch patients’ association, and by holding regular meetings, and have Dr. XX participate in the patients’ association.” (Participant A).

#### Managing the medical conditions of HAM/TSP.

People with HAM/TSP manage their medical condition by monitoring and taking care not to worsen their condition. “Confirming medical conditions of HAM by blood tests” was a way for participants to get to know their medical condition. In addition, participants had practiced efforts “Taking care not to worsen the medical condition,” according to their own approach.

“I was told to have a blood sample taken once or twice a year. I was told that I should have my blood drawn because of the risk of developing leukemia.” (Participant D).

“(Doctor recommended) lactic acid bacteria drink. I drink two bottles of that day: morning and evening.” (Participant G).

#### Self-determination regarding treatment.

Although no curative treatment for HAM/TSP has been established, the participants were determined to undertake “Receiving drug treatment” or “Making the choice not to receive drug treatment.” Even after opting to undergo “Receiving drug treatment,” they sometimes reverted to “Making the choice not to receive drug treatment” regarding the use of other medications or the continuation of the same treatment. “Self-evaluating one’s choices regarding treatment” is another action that pertains to self-determination regarding treatment.

“When I was first diagnosed with HAM/TSP, I was told that the treatment would be steroids, but I felt resistant to it, so I did not take them at that time.” “I think it was necessary, in looking back, to receive steroid pulse therapy every couple of years to keep the viral load from increasing too much, to the point where the symptoms would progress.” (Participant C).

#### Concern about the impact of HTLV-1 infection on close relatives.

As HAM/TSP is an infectious disease that is caused by HTLV-1, one of the self-management strategies for their chronic conditions involves “Concern about the impact of HTLV-1 infection on close relatives,” such as informing a close relative of the possibility of their becoming infected with HAM/TSP or checking the results of HTLV-1 antibody tests on them.

“I thought it was inappropriate to verbally tell her, so I gave her this (a document summarizing my medical history). I did not want her to be upset, so I told her that the HTLV-1 antibody test results would be positive.” (participant E)

### Moving forward as people with HAM/TSP

Although the diagnosis of HAM/TSP forced participants to confront the realities that HAM/TSP brings, they moved forward as people with HAM/TSP and engaged in internal self-management by supporting each other and accepting the reality of living with HAM/TSP.

#### Supporting each other among people with HAM/TSP.

One strategy for treating chronic conditions is for patients with HAM/TSP to support others with the same illness. “Digesting negative emotions with people with HAM/TSP” refers to assimilating each other’s negative feelings induced by HAM/TSP, whereas “Supporting people with HAM/TSP as a fellow patient” refers to supporting their fellow patients on various aspects, such as involvement in patient-association-related activities and personal advice and encouragement.

“By helping them think through their problems together, I told them that they were not alone, and that if they came to the patients’ association anytime and talked with others, they would find many people who would understand their suffering.” (Participant B).

#### Accepting the reality of what HAM/TSP brings.

The people with HAM/TSP accepted the reality brought about by HAM through their coping behaviors of “Accepting living with HAM/TSP positively” and “Not resisting the reality that HAM/TSP brings.”

“But I think that I was chosen in this way, because the incidence rate of HAM/TSP is only 0.3%. Therefore, I think that I was chosen. So I think I have to use this as a weapon against myself.” (Participant A).

“I must let things happen as they happen. There’s no point in thinking about it.” (Participant D).

## Discussion

In the present study, we found 6 strategies for managing the chronic conditions caused by HAM/TSP. These strategies address the needs of patients with HAM/TSP to self-manage their unique chronic conditions. In an overview of the 6 core categories and 14 categories describing self-management strategies, three characteristics of HAM/TSP – lack of an established curative treatment, low social awareness of the disease, and its infectious nature – had a significant impact on the self-management of individuals with HAM/TSP.

Medication management is an important component of self-management [13]. In neurological disorders, taking medications is an important self-management strategy to follow the treatment regimen, and Audulv et al. have identified “Managing medication and treatment plan” as a sub-domain of the domain “Disease controlling strategies” in the self-management strategies of individuals with neurological conditions in the TEDSS [12,16]. However, for people with HAM/TSP who do not have an established curative treatment, medication adherence was not a priority self-management strategy of “Taking control of HAM/TSP.” Corticosteroids and IFN-α are the main treatments for HAM/TSP-induced inflammation in the spinal cord at the present stage [21]. All but one participant in this study received one of these therapies, although some participants chose not to continue treatment after receiving the treatment once. One participant regretted not receiving treatment during the early stages of HAM/TSP after the diagnosis. As treatments may require hospitalization and potentially have side effects, the decision to receive medication is a consideration for people with HAM/TSP. “Self-determination regarding treatment,” under the core category of “Taking control of HAM/TSP,” has become a self-management strategy for individuals with HAM/TSP in the context of their medical condition and social situation. Regarding medication management, two participants were taking muscle relaxants for lower-limb spasticity, and three participants were taking medications such as alpha-1 blockers for urinary dysfunction. However, the study results did not identify the use of these medications as a self-management strategy. For participants, maintaining motor function through rehabilitation or finding their own ways to manage residual urine or urinary incontinence may have been readily recognizable self-management strategies. However, it cannot be denied that taking muscle relaxants and medication for urinary dysfunction creates a certain stable state that helps maintain walking and urinary functions. Therefore, we consider it necessary to explore self-management strategies thoroughly for medication management regarding symptomatic treatment going forward.

In addition, individuals with HAM/TSP engaged in the self-management strategy of “Ensuring a specialized treatment environment for HAM/TSP,” which may be a characteristic type of “Disease controlling strategy” for people with HAM/TSP for whom no curative treatment has been established. In the absence of a cure, it is important to ensure that the treatment environment for people with HAM involves not only “Ensuring medical treatment by HAM/TSP specialist” but also “Supporting research on the treatment of HAM/TSP.” Besides the strategy of “Taking control of HAM/TSP,” the identification of a strategy for “Ensuring a specialized treatment environment for HAM/TSP” is considered one of the characteristics of self-management among people with HAM/TSP.

In the results of this present study, “Attempting to maintain body functions,” and “Acquiring new ways to complement body functions” were important self-management strategies for HAM/TSP-affected body functions. For people with HAM/TSP for whom no curative treatment has been established, the “Management and prevention of symptoms and complications,” which was a sub-domain of “Disease controlling strategies” in the TEDSS [12,16], constitutes an important self-management strategy. Individuals with HAM/TSP achieved prevention, control, and limitation of symptoms, complications, and disease progression through self-management strategies, such as controlling impaired excretory function in unique ways, introducing self-catheterization to prevent urinary tract infection owing to residual urine retention, and training to maintain motor function. Furthermore, individuals with HAM/TSP attempted to control the disease’s impact on daily life and maintain their previous routines through a self-management strategy of “Engaging to maintain an independent daily life alongside the “Taking control of HAM/TSP” strategy. The participants attempted to become agents of their own control over the physical functions, symptoms, and daily lives that are affected by HAM/TSP. Bishop et al. found that self-management is strongly associated with perceived control and that both perceived control and self-management mediate the relationship between the impact of MS and the subjective quality of life among individuals with MS [22]. Thus, enabling people with HAM to manage and prevent symptoms and complications, accompanied by the perception that they are in control of the effects of HAM/TSP, may be an important self-management strategy that affects their subjective quality of life.

The lack of public awareness of HAM/TSP necessitates the incorporation of self-management strategies right from the time that an individual is diagnosed with HAM/TSP. For a common disease, a diagnosis provides answers to the current and future conditions. On the one hand, for people with HAM/TSP, strategies are needed to understand the situation caused by the unfamiliar with HAM/TSP. “Acting to understand HAM/TSP” within the core category “Taking control of HAM/TSP” would be characterized as a preparatory and proactive strategy for people with HAM/TSP to confront their potential chronic conditions and make decisions about how they will cope with their HAM/TSP in the future. On the other hand, the lack of awareness of HAM/TSP is similar in medical settings, and “Ensuring a specialized treatment environment for HAM/TSP” is an important self-management strategy for people with HAM/TSP. Coler-Reilly et al. conducted an epidemiological study of HAM/TSP in Japan by using a registry system and found that the median duration from onset to diagnosis of HAM/TSP was 5 (range, 1–12) years [23]. The limited awareness of HAM/TSP among general physicians in HTLV-1-non-endemic areas in Japan makes it difficult for individuals with HAM/TSP to reliably receive an accurate diagnosis. Furthermore, Zihlmann et al. pointed out that primary healthcare teams are generally unaware of HTLV-1 and do not recognize the health problems associated with the infection and consequent disease; this gap creates uncertainty regarding the medical knowledge of the team [24]. Early diagnosis and medical treatment by HAM/TSP specialists are indispensable for patients with HAM/TSP, as it affects disease progression and prognosis. Therefore, “Ensuring a specialized treatment environment for HAM/TSP” can be an important self-management strategy that also affects “Attempting to maintain body functions,” “Engaging to maintain an independent daily life,” and “Taking control of HAM/TSP.” The Comprehensive Measures for HTLV-1 was endorsed by the Japanese government in 2010 and the medical system in Japan is relatively well developed [25]. However, as participants in this study mentioned, survey results indicate that people with HAM/TSP are forced to bear the burden of receiving highly specialized and effective treatment, such as commuting several hours to see a specialist [26]. “Ensuring a specialized treatment environment for HAM/TSP” is an essential self-management strategy for people with HAM/TSP; however, there are limits to what individuals with HAM/TSP can manage on their own, and social challenges remain.

Furthermore, the infectious nature of HAM/TSP affects the self-management strategies. Individuals with HAM/TSP must consider the effects of HTLV-1 not only on themselves but also on their close relatives. This self-management strategy is specific to people with HAM/TSP and is not found in other neurological disorders. The results of the present study identified as a category of “Concern about the impact of HTLV-1 infection on close relatives” within the core category “Taking control of HAM/TSP.” One participant was concerned not only about transmission to close relatives, but also that if a close relative was an HTLV-1 carrier, he or she could spread HTLV-1 infection through vertical transmission, breastfeeding, and sexual intercourse, or limit those activities. This characteristic self-management strategy of people with HAM/TSP may include two intentions. The first is the proactive intention to prevent further spread of HTLV-1 infection through infection prevention actions. Preventing the spread of HTLV-1 reduces the risk of HAM/TSP and other related diseases for close relative. The second aim was to minimize misconceptions about HTLV-1 infection and its associated impact on relationships with others and social life. Zihlmann et al. found that many of their HTLV-1-infected participants tended to believe that their disease was an HIV infection or were in denial that they had HTLV-1, and that fear of exposing their infection and the destruction of family relationships that a diagnosis of HTLV-1 might cause led them to treat their diagnosis as a “secret” [24]. Davoudi et al. found that people with HAM/TSP, when they came to know of their HTLV-1 infection, experienced an impact on their desirable personal and social lives, as the fear of spreading the virus prevents them from continuing their marital lives and may even lead to divorce [17]. Although some of the participants’ narratives in the present study expressed concern about the roots of their HAM/TSP, exploring the roots of their HTLV-1 infection can cause family discord and confer a psychological burden for people with HAM/TSP if there is no proper understanding of HTLV-1 infection. Thus, HAM/TSP is prone to social stigma owing to low social awareness of the disease and the fact that it is an infectious disease; thus, self-management strategies are required to minimize this impact. Within the core category “Engaging to maintain an independent daily life” of self-management strategies identified in this study for people with HAM/TSP, “Receiving the required support” may be difficult for people with HAM/TSP to openly receive the support that they need in a society where HAM/TSP is stigmatized and precludes openness about suffering from HAM/TSP; this may affect the social and psychological self-management strategy “Moving forward as people with HAM/TSP.” In Japan, the public initiatives of Comprehensive Measures for HTLV-1 include infection-prevention measures, such as the inclusion of HTLV-1 antibody testing in antenatal pregnancy screening, and measures to increase awareness and provide information, such as the website of the Ministry of Health, Labor for providing information on HTLV-1 infection and associated diseases, and published materials to increase public awareness [26]. Although measures at the national and municipal levels are being expanded, the concerns and hardships for individuals with HAM/TSP remain endless.

Finally, we suggest care approaches that we consider useful for patients with HAM/TSP. Nurses will need to implement personalized educational sessions to help individuals with HAM/TSP develop the skills to self-manage the impact of HAM/TSP on their body functions, and thereby enable them to experience control over their HAM/TSP. We believe that the supportive role of nurses could help patients with HAM/TSP recognize that their disease is under their control and enable the identification of individualized strategies for symptom management and prevention of complications. Moreover, nurses may need to undertake self-management advocacy to help patients with HAM/TSP fully understand and make decisions regarding their treatment and participation in clinical trials, including understanding their personal preferences, coordinating with their physicians, and providing the information necessary for decision-making. Furthermore, we believe that the self-management strategy of “Concern of the impact of HTLV-1 infection on close relatives” within the core category “Taking control of HAM/TSP” should never be left to the patient alone. Nurses may need to play a role not only in providing more accessible support to people with HAM/TSP and those around them, such as guidance and counseling on understanding HTLV-1 and imparting information on correct infection-control measures, but also in national policies from the nurses’ perspective, ensuring that the dignity of people with HAM/TSP is preserved.

## Limitations

An important limitation of this study was that, owing to the background of recruiting participants from HAM/TSP patient associations and the small sample size, the study’s results may not describe all aspects of self-management in people with HAM/TSP. We were forced to collect data from the recruited participants, and this made it difficult to conduct theoretical sampling. Although we were unable to increase the number of cases for saturation, we were able to identify an overview and variations in the self-management strategies that were analyzed for different cases, and we consider it worth reporting as a basic research finding in this field. Further studies are needed for a detailed exploration of each of the self-management strategies of HAM/TSP by expanding the survey to include those who were not members of the HAM/TSP patient association.

## Conclusion

This study explored the self-management strategies of individuals with HAM/TSP in Japan. Consequently, 6 core categories were identified in self-management strategies for managing the chronic HAM/TSP-induced conditions. The self-management strategies of individuals with HAM/TSP had a significant impact from the 3 characteristics of HAM/TSP: lack of an established curative treatment, low social awareness of the disease, and its infectious nature. Personalized education to develop self-management skills in individuals with HAM/TSP and accessible support to prevent patients from struggling alone are needed.
